# Numerical evaluation of a radiant panel system for heating a high-ceiling room

**DOI:** 10.1186/2046-7648-4-S1-A156

**Published:** 2015-09-14

**Authors:** António M Raimundo, A Virgílio M Oliveira, Adélio R Gaspar, Divo A Quintela

**Affiliations:** 1 ADAI-LAETA, Department of Mechanical Engineering, University of Coimbra, Coimbra, Portugal; 2 Coimbra Institute of Engineering, Polytechnic Institute of Coimbra, Department of Mechanical Engineering, Coimbra, Portugal

## Introduction

It is common to assess the level of wasted energy due to the inappropriate selection of HVAC systems (Heating, Ventilation and Air Conditioning) that are regularly used to control temperature in man-made environments. The present work investigates the use of radiant panels as alternative method for controlling the thermal environment and aims to contribute to the characterization of the performance of these devices. It is a technology that has already proven its effectiveness in terms of cooling, however, still little explored for heating, particularly in spaces with high ceilings.

## Methods

A numerical methodology was used for evaluating the convective and radiative heat exchanges between the human body and the environment and surrounding surfaces. 3D-CFD software was used (Computational Fluid Dynamics), enhanced with a radiation module, both developed by the authors. The radiation module accounts for the radiative heat transfer between the active surfaces, including those related to the occupants. This module comprises two calculating stages: a pre-processor and an inner block in the CFD code. The pre-processor calculates the heat exchange of actively radiating areas of each participating surface and the Gebhart absorption factors, where the shading effects were taken into account and for each iteration of the CFD code, the inner block checks the radiative heat exchanges between all the surfaces.

## Results

For a selected room with a high-ceiling (11.8 × 8.6 × 6.7 m), under cold climatic conditions, the CFD software was used to obtain the flow structure, temperature field, the thermal comfort in the human occupied zone and the energy consumption by the HVAC system. Three different room-conditioning strategies were analysed: (*i*) without a heating system (to serve as reference); (*ii*) radiating heating panels occupying 30% of the ceiling area; and (*iii*) a traditional system based on the supply of heated air.

## Discussion

For the situation without a heating system, the global human body sensible heat losses were 152.85 W, 67.31 W by convection and 85.54 W by radiation, for which *PMV *= -1.01 (ISO 7730, 2005) [1], corresponding to a subjective feeling of moderate cold. With the radiating panels at 90 °C, the global human body sensible heat losses were 93.92 W, 71.14 W by convection and 22.78 W by radiation, for which *PMV *= - 0.04, corresponding to a feeling of thermal comfort. Under this condition the panels' energy stream is about 45.5 W per m^2 ^of room floor. To achieve a similar *PMV *value with the traditional system based on the supply of heated air about 6 times this amount of energy is needed. In addition, the air velocity promoted by a standard HVAC system is very high, promoting local thermal discomfort by draught.

## Conclusion

The results demonstrate the suitability of systems based on radiant panels for heating spaces with high ceilings because they promote a significant decrease of the radiation losses by the human body, which is positive in terms of thermal comfort. When compared with a traditional heating system based on fan coil units, the radiating panels system is more effective in terms of thermal comfort and energy efficiency.

## References

[B1] ISO 7730 Ergonomics of the thermal environment - Analytical determination and interpretation of thermal comfort using calculation of the PMV and PPD indices and local thermal comfort criteria 2005 International Organization for Standardization, Genève

